# Proportion of Late Pregnant Women with Anxiety Symptom under COVID-19 Pandemic in Japan

**DOI:** 10.31662/jmaj.2021-0204

**Published:** 2021-12-24

**Authors:** Ryuhei Kurashina, Shunji Suzuki

**Affiliations:** 1 Department of Obstetrics and Gynecology, Japanese Red Cross Katsushika Maternity Hospital, Tokyo, Japan

**Keywords:** anxiety, pregnancy, COVID-19 pandemic, the two-item generalized anxiety disorder scale (GAD-2), parity, Japan

## Abstract

**Introduction:**

The COVID-19 pandemic has caused stress and anxiety for pregnant women worldwide. We examined the anxiety symptom in Japanese women during pregnancy using a self-administered questionnaire under the COVID-19 pandemic.

**Methods:**

Between April 2020 and March 2021 (2020, the COVID-19 pandemic), we asked 248 Japanese women without history of mental disorders who delivered singleton neonates at 37-41 weeks’ gestation to answer the two-item generalized anxiety disorder scale (GAD-2) at first, second, and third trimesters of gestation. We also asked 311 women with the same situation between January 2019 and December 2019 (2019) as control.

**Results:**

The women with anxiety symptom were common during the first trimester of gestation irrespective of COVID-19 pandemic. In 2019, the proportion of the women with anxiety symptom decreased as the trimester of pregnancy progressed (*p* < 0.01); however, in 2020, the proportion of women with anxiety symptom did not decrease during pregnancy. During the late pregnancy, the proportions of women with anxiety symptom in 2020 were significantly higher than those in 2019 (*p* < 0.01) regardless of maternal parity or age in Japan.

**Conclusion:**

The COVID-19 pandemic seemed to prevent the decrease in anxiety symptom that should decrease as pregnancy progresses regardless of maternal parity or age in Japan.

## Introduction

Recently, perinatal anxiety disorder has been one of the most common mental health problems in pregnant women, associated with increased risks of adverse perinatal outcomes and recognized as serious safety problem ^(1), (2)^. Although it is normal to feel a little worried and stressed during pregnancy, for some people, anxiety can become a real problem. The symptom levels of anxiety and/or stress have been observed to change during gestation ^(3), (4)^. As many as 30% of pregnant women will experience some various anxieties, and a few women may have more severe symptom and will be diagnosed with an anxiety disorder ^(2)^.

During gestation in Japan, the levels of general and maternal anxiety have appeared to be high at the first trimester and decrease as the pregnancy progresses ^(5), (6)^, because women during the first trimester of gestation will have many anxieties such as fear of miscarriage, morning sickness, and abnormal childbirth. Moreover, an increased progesterone will affect their emotions and increases anxiety. For example, in our earlier study in 2017, the incidence of anxiety symptom was observed to be in 36% of the women during the first trimester of pregnancy, while the rate was decreased to be 13 and 7% during the second and third trimester of pregnancy, respectively ^(7)^.

The COVID-19 (coronavirus disease 2019) pandemic has caused stress and anxiety for pregnant women worldwide ^(8)^. Some previous observations could be used to develop appropriate strategies for the management of mental health problems during gestation under the COVID-19 pandemic ^(9), (10)^. Our recent observation may indicate that the mental status of Japanese women is unstable due to various physical and/or social changes during the first trimester of gestation under the COVID-19 epidemic ^(11)^.

Based on these backgrounds, we examined the anxiety symptom in Japanese women during pregnancy using a self-administered questionnaire under the COVID-19 pandemic.

## Materials and Methods

The protocol for this study was approved by the Ethics Committee of Japanese Red Cross Katsushika Maternity Hospital (2021-14). From all pregnant women, informed consent concerning retrospective analyses was obtained.

Between April 2020 and March 2021, we asked 250 Japanese women without history of mental disorders who delivered singleton neonates at 37-41 weeks’ gestation at our institute to answer the two-item generalized anxiety disorder scale (GAD-2) at first, second, and third trimesters of gestation (8-11, 20-24, and 34-37 weeks of gestation), and 248 women (99%) gave us analyzable answers. The GAD-2 is a self-administered questionnaire for generalized anxiety disorder. The questions are as follows: (1) how often have you been bothered by feeling nervous, anxious, or on edge over the last 2 weeks? (2) How often have you been bothered by not being able to stop or control worrying over the last 2 weeks? If at least one of the two questions is “yes,” we evaluated the woman as having anxiety symptom. In this study, we excluded the women with mental disorders who were already receiving medication and/or psychotherapy. In this study, we also asked 315 women with the same situation between January 2019 and December 2019 as control, and 311 women (99%) gave us analyzable answers.

Using medical charts of the women who responded validly, we examined parity, maternal age, and history of mental disorders. In addition, we examined the relation between the results of the GAD-2 and the maternal parity and age (≥35 years).

The results are expressed as number (%). Significant differences (*p* < 0.05) were determined by one-way analysis of variance and the *Χ^2^* test. The odds ratio (OR) and 95% confidence interval (CI) were calculated.

## Results

There were no significant differences in maternal age or parity between the women in 2019 and 2020 as shown in Table 1.

**Table 1.** Clinical Characteristics of the Women in 2019 and 2020.

Year	2019	2020	*p*-value
	Control	COVID-19 pandemic	
Total number	311	248
Nulliparous women	195 (63)	168 (68)	0.22
Maternal age			
<20 years	7 (2)	4 (2)	0.59
≥35 years	111 (36)	101 (41)	0.11

Data are presented as number (%)

Table 2 shows the results of the GAD-2. The women with anxiety symptom were common during the first trimester of gestation irrespective of COVID-19 pandemic. In 2019, the proportion of the women with anxiety symptom decreased as pregnancy progressed (*p* < 0.01); however, in 2020, the proportion of women with anxiety symptom did not decrease during pregnancy. During the second and third trimesters of gestation, the proportions of women with anxiety symptom in 2020 were significantly higher than those in 2019 (*p* < 0.01) as shown in Table 2.

**Table 2.** Results of Answers for the Generalized Anxiety Disorder Scale-2 (GAD-2) during Pregnancy in 2019 and 2020.

Year	2019	2020	*p*-value	OR	95% CI
	Control	COVID-19 pandemic			
Total number	311	248			
First trimester	105 (34)	79 (32)	0.63		
Second trimester	37 (12)*	65 (26)	<0.01	2.63	1.7-4.1
Third trimester	22 (7)*	60 (24)	<0.01	4.19	2.5-7.0

Data are presented as number (%) OR, odds ratio 95% CI, 95% confidence interval *p < 0.05 vs. first trimester

As shown in Table 3, the anxiety symptom during the third trimester of pregnancy was associated with nulliparity (OR 6.51, 95% CI 1.7-2.6, *p* < 0.01) in 2019; however, they were not associated with parity or maternal age in 2020 as shown in Table 4.

**Table 3.** Clinical Characteristics and Results of Answers for the Generalized Anxiety Disorder Scale-2 (GAD-2) during the Third Trimester of Pregnancy in 2019.

Result of the GAD-2	Positive	Negative	*p*-value
Total	22	289
Nulliparity
Yes	20 (91)	175 (61)
No	2 (9)	114 (39)	<0.01
Maternal age ≥ 35 years
Yes	12 (55)	99 (52)	
No	10 (45)	90 (48)	0.06

GAD-2, generalized anxiety disorder scale-2 Data are presented as number (%)

**Table 4.** Clinical Characteristics and Results of Answers for the Generalized Anxiety Disorder Scale-2 (GAD-2) during the Third Trimester of Pregnancy in 2010 (COVID-19 Pandemic).

Result of the GAD-2	Positive	Negative	*p*-value
Total	60	188
Nulliparity
Yes	41 (68)	107 (57)
No	19 (32)	81 (43)	0.12
Maternal age ≥ 35 years
Yes	22 (37)	79 (42)
No	38 (63)	109 (58)	0.46

GAD-2, generalized anxiety disorder scale-2 Data are presented as number (%)

## Discussion

Based on the rapid review assessed in 34 studies in 2021 ^(12)^, the pooled prevalence of anxiety during gestation was 30.5%. The prevalence of anxiety seemed to be higher in studies conducted later in the pandemic. The COVID-19 pandemic had a profound effect on the mental health of pregnant women, and factors unrelated to gestation appeared to be causing pregnancy-specific changes in anxiety ^(13)^. In particular, high-risk pregnancy patients most often had comorbidities, which increased not only their risk of infection but also their anxiety scores due to the stress caused by COVID-19 pandemic ^(14)^. The risk of depression and anxiety increases with high-risk pregnancies ^(15)^. In a recent study in Italy under the COVID-19 pandemic, 47, 65, and 51% of pregnant women had anxiety symptom due to fear over potential fetal anomalies, fear over fetal growth restriction, and fear over premature delivery caused by COVID-19, respectively ^(16)^. Our institute is one of the main perinatal centers in Tokyo primarily for high-risk pregnancies. Therefore, the current results may support the previous studies ^(14), (15), (16)^. It may be necessary to pay more attention to the mental status of pregnant women under the pandemic ^(17)^. It will be recommended to reduce anxiety caused by coronavirus and pregnancy concerns.

In earlier studies without COVID-19 pandemic, the anxiety symptom during gestation has been reported to be associated with nulliparity and/or advanced maternal age due to worry about self and/or baby concerns ^(18), (19)^. Their generally mild anxiety has been reported to be associated with their having no experience of delivery or labor pain. The relation between anxiety during pregnancy and nulliparity under the COVID-19 pandemic has been variable ^(20), (21), (22), (23)^. Awad-Sirhan et al. ^(20)^ reported the having more than one child as a specific risk factor for anxiety in pregnant women under the COVID-19 pandemic in Spain those requiring pregnancy care and follow-up protocols. Effati-Daryani et al. ^(21)^ also observed a low level of anxiety in nulliparous women during the outbreak of COVID-19 in Iran. Some other results have indicated that nulliparity is one of the predictors of women’s worries under the COVID-19 pandemic ^(22), (23)^. Women who get pregnant for the first time may be in difficult periods due to both the threat of the infection and the discomforts and complications of gestation. However, in the present study, the COVID-19 pandemic itself made women in late gestation uneasy regardless of maternal parity or age in Japan.

Reliable information on pregnancy and its complications under to the COVID-19 pandemic is scarce. However, according to previous epidemics of SARS and MERS, which were associated with several physical and psychological changes during gestation, pregnant women are more susceptible to the virus. Physiological changes in gestation will also lead to psychological problems and disruption of their sociological roles ^(24)^. Consequently, these changes can cause some problems such as emotional instability, stress, and anxiety in pregnant women ^(25)^. Social distance-related restrictions that interfere with communication with relatives, friends, and others will also increase stress and anxiety in daily life ^(26)^. Fortunately, the number of pregnant women infected with coronavirus has been lower than that of ordinary women in Japan; however, some various social and/or psychological factors have seemed to stimulate the anxiety of pregnant women ^(27), (28)^.

In the present study, some serious limitations will exist besides the small sample size. During the study period, we did not examine the mental status of non-pregnant women as a control group, so the effects of pregnancy have not been fully investigated. In our institute, the GAD-2 has been used to support the mental health of our pregnant women; however, we understand that the GAD-2 is just a screening tool that cannot make a definitive diagnosis of anxiety disorders. In our institute, we have used the GAD-2 to avoid overlooking pregnant women who need social or mental support only. Therefore, this study may not be definitive for the proportion of anxiety in pregnancy.

However, in conclusion, the COVID-19 pandemic seemed to prevent the decrease in anxiety symptom that should decrease as pregnancy progresses.

## Article Information

### Conflicts of Interest

None

### Author Contributions

RK and SS designed the study. SS collected the data. RK and SS analyzed the data. RK wrote the first draft. RK and SS authors have accepted responsibility for the entire content of this manuscript and approved its submission.

### Approval by Institutional Review Board (IRB)

The protocol for this study was approved by the Ethics Committee of Japanese Red Cross Katsushika Maternity Hospital (2021-14). From all subjects, informed consent concerning retrospective analyses was obtained. The data supporting the results of the present study are available within the article.
